# Detecting Enclosed Board Channel of Data Acquisition System Using Probabilistic Neural Network with Null Matrix

**DOI:** 10.3390/s22155559

**Published:** 2022-07-25

**Authors:** Dapeng Zhang, Zhiling Lin, Zhiwei Gao

**Affiliations:** 1 School of Electrical and Information Engineering, Tianjin University, Tianjin 300072, China; zdp@tju.edu.cn; 2 School of Electrical Engineering, Tianjin University of Technology, Tianjin 300384, China; 3 Faculty of Engineering and Environment, University of Northumbria, Newcastle upon Tyne NE2 8ST, UK; zhiwei.gao@northumbria.ac.uk

**Keywords:** fault detection and diagnosis, board channel, probabilistic neural network

## Abstract

The board channel is a connection between a data acquisition system and the sensors of a plant. A flawed channel will bring poor-quality data or faulty data that may cause an incorrect strategy. In this paper, a data-driven approach is proposed to detect the status of the enclosed board channel based on an error time series obtained from multiple excitation signals and internal register values. The critical faulty data, contrary to the known healthy data, are constructed by using a null matrix with maximum projection and are labelled as training examples together with healthy data. Finally, the status of the enclosed board channel is validated by a well-trained probabilistic neural network. The experimental results demonstrate the effectiveness of the proposed method.

## 1. Introduction

Data acquisition systems play a vital role in the data collection of industry [1]. Among them, the board tunnel, which is usually classified as analog input (AI), analog output (AO), digital input (DI), and digital output (DO) modules, is a bridge between the processor and sensors, which ensures the data conversion at the physical level [2]. The tunnel board is made up of enclosed circuit boards that are convenient to be replaced immediately once they are found to have any faults occur due to security reasons. In order to detect the inertial faults of these circuit boards in time, most famous products, such as Siemens, Honeywell, etc., have provided error codes to help operators [3,4,5]. However, these codes are limited to meeting the requirements of board channel diagnosis in a practical complex application.

Different kinds of methods for fault detection and diagnosis (FDD) have been developed, which are classified as model-based approaches, signal-based approaches, and data-driven approaches [6,7]. In model-based approaches, the fault diagnosis algorithms are developed to monitor the consistency between the measured outputs of the practical systems and the model-predicted outputs, which are based on an appropriate model, whether a physical model or equivalent model. Reference [8] proposed a new method by combining the model-based FDD method and the support vector machine (SVM) method. In reference [9], the spindle modes are determined through a three-step procedure in order to overcome these issues of the low number of sensors and the presence of many harmonics in the measured signals and to extract the characteristics of the system. In reference [10], based on the information of fault-free data series, fault detection was promptly implemented by comparison with the model forecast and real-time process. Signal-based approaches include time-domain analysis, frequency-domain analysis, and both together. Reference [11] proposed a novel “frequency-domain damping design” using a high-pass filter for acceleration-based bilateral control (ABC) based on modal space analysis. In reference [12], a unified measurement model was utilized to simultaneously characterize both the phenomena of multiple communication delays and data missing, and a novel residual matching (RM) approach was developed to isolate and estimate the fault once it is detected. Reference [13] proposed a least squares support vector machine (LS-SVM) model optimized by cross validation to implement FDD on a 90-ton centrifugal chiller. Reference [14] investigated the achievable rates of frequency-division-duplex massive MIMO systems with spatially correlated channels. In fact, it is difficult for the board tunnel to build an appropriate model since different boards have different circuit structures. It is also a challenge to obtain the features of integer signals, especially for the fault cases, because the flawed board tunnel will be quickly replaced for safety reasons.

Board tunnels always work on a standard enclosed module, which prevents the circuit from being affected by external factors. This enclosed module is also suitable for quick disassembly or replacement. However, as a double-edged sword, this method introduces issues for fault detection and diagnosis because it loses the ability to directly observe internal states. The data-driven approach [15,16,17,18,19,20] provides a feasible way to solve this problem by external observation data. Reference [15] aimed to provide a state-of-the-art overview on the existing fault diagnosis, prognosis, and resilient control methods and techniques for wind turbine systems, with which great success has been achieved in fault detection and diagnosis. Reference [16] focused on data-driven techniques in the digital era and data analytics in all areas, including process industries. Reference [17] proposed a new data-driven FDD method, which was named probability-relevant PCA (PRPCA), for electrical drives in high-speed trains. In reference [18], a fault diagnosis method based on a deep convolutional neural network model consisting of convolutional layers, pooling layers, dropout, and fully connected layers was proposed for chemical process fault diagnosis. In reference [19], an extended deep belief network (EDBN) was proposed to fully exploit useful information in the raw data. Reference [20] presented a Special Issue on “data-driven approaches for complex industrial systems”. Using a data-driven approach to the board tunnel detection, two obstacles should be overcome: (1) A healthy board shows certain differences in response to the process conditions, working environment, and internal parameters. This dispersivity is difficult to be covered by limited sample data. (2) Generally, the stability of a data acquisition system is generally high, and there are few failures; even if a failure occurs, it will be replaced quickly in order to achieve safety. Therefore, there are almost no historical faulty data.

From the view of board performance, the healthy data will obey the law of health probability distribution, though the healthy data are dispersive in different working environments. Some excellent methods based on probability analysis, such as the conditional probability distributions, Bayesian network, etc., have been reported in chemical processes [20,21,22,23]. Motivated by the probability idea based on the concept that the acquired data signal is regarded as a realization of the distribution of the board, a probabilistic neural network (PNN) is proposed based on critical faulty data being artificially constructed to distinguish between healthy states and faulty states. Firstly, multiple data sources are applied to activate conditions on the board tunnel, and the internal register values are obtained by OPC technology. Then, the error time series are constructed to analyze the healthy state of the enclosed board channel. The critical faulty data are constructed based on the healthy data by using a null matrix with maximum projection. Finally, the healthy state of the enclosed board channel is judged by a probabilistic neural network. The advantages of the proposed approach are summarized as follows:

(1) Multiple input signals are proposed to activate the working state of the board tunnel, which extends the scope of exploration for the dispersivity of a healthy board concerning the working environment and internal parameters.
(2) The critical faulty data are successfully constructed by using the null matrix based on the health data, which overcomes the difficulty of lacking faulty data.
(3) The PNN is used to adapt to the law of probability hidden in the time series, and case studies verify the effectiveness.

The remainder of this article is organized as follows. In Section 2, the acquisition of error time series and the relationship between multiple input signals and overall performance of the board tunnel are given. Section 3 describes the proposed approach, including the probability neural network, the construction of critical faulty data, the structure, and the workflow. The case studies are illustrated in Section 4, followed by conclusions in Section 5.

## 2. The Error Time Series of Board Tunnel

**Figure 1:**
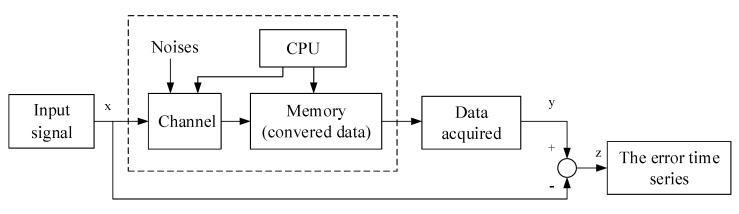
The acquisition principle of error time series with a single input signal.

The error between input signal and output (memory) mainly affected by internal factors of the board is regarded as a comprehensive index reflecting the performance of the board tunnel. A single sample is meaningless for evaluating the board performance because it is an instance and not enough to observe the law of probability. Thus, an error time series is taken as an analysis object of the enclosed board tunnel, and the error time series is obtained, as shown in Figure 1.

Let the input signal of the board tunnel be ${\left\{x\left(k\right)\right\}}_{k=1}^{\infty }$ and the value of the corresponding memory be ${\left\{y\left(k\right)\right\}}_{k=1}^{\infty }$; thus, the error time series is

(1) $${\left\{z\left(k\right)\right\}}_{k=1}^{\infty }={\left\{y\left(k\right)\right\}}_{k=1}^{\infty }-{\left\{x\left(k\right)\right\}}_{k=1}^{\infty }$$

where is the sampling time. Formula (1) is abbreviated as Formula (2) by using *x, y, z* instead of ${\left\{x\left(k\right)\right\}}_{k=1}^{\infty }$,${\left\{y\left(k\right)\right\}}_{k=1}^{\infty }$,${\left\{z\left(k\right)\right\}}_{k=1}^{\infty }$.

(2) z=y−x

Notice that is the converted data of input signal *x* according to the physical meaning of the board channel, and *z* is regarded as a probability model of noisy influences that follows a normal distribution with a form of Formula (3):

(3) $$z∽N\left(\mu_{board},\sigma_{board}^{2}\right)$$

where $\mu_{board}$ and $\sigma_{board}$ are an expectation and a variance for the board, respectively.

It is worth noting that if the board input x is enough to cover all the work conditions and influences of the environment, the expectation $\mu_{board}$ is equal to the mean, which ideally satisfies$\left\{\begin{array}{c} \begin{array}{cc} \mu_{board}=0 & y=x \end{array}\\ \begin{array}{cc} \mu_{board}\neq 0 & y\neq x \end{array} \end{array}\right.$ . Thus, thereafter, we use the mean instead of the expectation.

**Figure 2:**
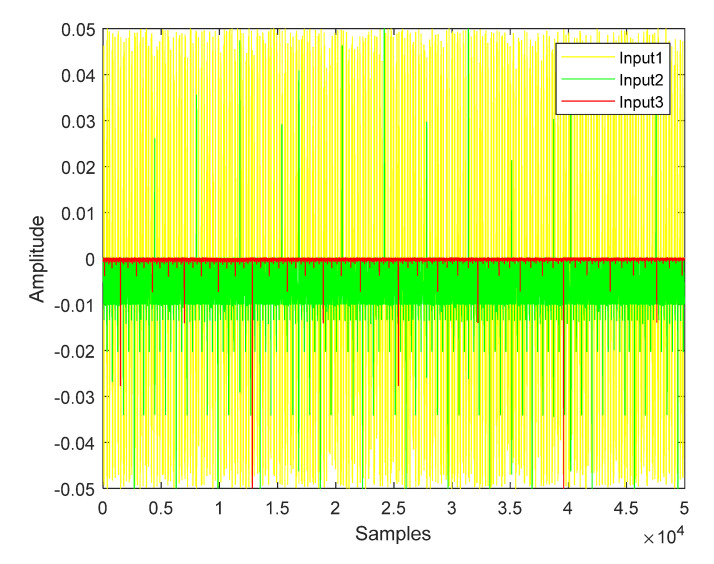
The error time series of different input signals under healthy status.

In fact, different input signals will cause some changes due to the influence of the environment and internal parameters. Figure 2 releases the error time series of a healthy board channel under three kinds of different input signals.

Each input signal that is long enough will produce its own probability distribution laws with a form of

(4) $$z_{i}∽N\left(\mu_{i},\sigma_{i}{}^{2}\right)$$

where *μ_i_* and *σ_i_* are the mean and the variance under the *i*-th input signal. It is inevitable for some deviations to occur between $\mu_{board}$ and $\mu_{i}$. From the view of fault detection and diagnosis, the board tunnel is considered to be in a healthy state as long as $\mu_{board}$ is within the allowable range. However, these deviations between $\mu_{board}$ and $\mu_{i}$ will disturb the judgment of healthy states due to the limitation of the sampling data number. In order to establish the relation between sampling data and board performance, it is assumed that the mean $\mu_{board}$ is equal to the mean of different input signals, that is,

(5) $$\mu_{board}=\frac{1}{n}\sum_{i=1}^{n}\mu_{i}$$

**Lemma** **1.** *The mean μ_board_ and variance* $\sigma_{board}^{2}$ *of the sampling data series* z *satisfying normal distribution can be replaced by* m *sub-sampling data whose mean is* $\mu_{m}\left(i\right),\;i=1,2,\cdots ,m$ *and whose variance is* $\sigma_{m}^{2}\left(i\right),\;i=1,2,\cdots ,m$ *. That is,*

(6) $$\mu_{board}=\frac{1}{m}\sum_{i=1}^{m}\mu_{m}\left(i\right)$$

(7) $$\sigma_{board}^{2}=\frac{1}{m-1}\sum_{i=1}^{m}\sigma_{m}^{2}\left(i\right)$$

**Proof.**  For the data series $z\sim N\left(\mu_{board},\sigma_{board}^{2}\right)$ that follows normal distribution with a mean $\mu_{board}$ and variance $\sigma_{board}^{2}$, suppose the data series z has enough data of n samples to reflect the statistical characteristics of a whole. The unbiased estimate of $\mu_{board}$ is $\bar{z}$, and the unbiased estimate of $\sigma_{board}^{2}$ is obtained according to

(8) $$\sigma_{board}^{2}=\frac{1}{n-1}\sum_{i=1}^{n}{\left(z_{i}-\bar{z}\right)}^{2}$$

□

Consider the relation of the mean between the whole and sub-sampling data. Let the n samples be divided into m groups with the mean and the length of the *k*-th group being ${\bar{z}}_{m}\left(k\right)$ and $L_{m}\left(k\right)$:

(9) $${\bar{z}}_{m}\left(k\right)=\frac{1}{L_{m}\left(k\right)}\sum_{i=1}^{L_{m}\left(k\right)}z_{i}$$

Thus, the mean of a whole is

(10) $$\frac{1}{m}\sum_{k=1}^{m}{\bar{z}}_{m}\left(k\right)=\frac{1}{m}\sum_{k=1}^{m}\frac{1}{L_{m}\left(k\right)}\sum_{i=1}^{L_{m}\left(k\right)}z_{i}=\frac{1}{\sum_{k=1}^{m}L_{m}\left(k\right)}\sum_{k=1}^{m}\sum_{i=1}^{L_{m}\left(k\right)}z_{i}=\frac{1}{n}\sum_{i=1}^{n}z_{i}=\bar{z}$$

Formula (10) shows the unbiased estimate of $\bar{z}$. Therefore, $\mu_{board}$ can be estimated by the above formula.

For a variance, it is well known that the sample mean of normal distribution also obeys normal distribution according to the mathematical statistical theory. Thus, the mean ${\bar{z}}_{i}$ of each group follows

(11) $${\bar{z}}_{i}∽N\left(\mu_{i},\frac{\sigma_{board}^{2}}{L_{m}}\right)$$

Let $\sigma_{i}^{2}=\frac{\sigma_{board}^{2}}{L_{m}}$; thus, ${\bar{z}}_{i}∽N\left(\mu_{i},\sigma_{i}^{2}\right)$.

For m groups, an unbiased estimate of $\sigma_{i}^{2}$ is obtained by

(12) $$\sigma_{i}^{2}=\frac{1}{m-1}\sum_{k=1}^{m}{\left({\bar{z}}_{i}\left(k\right)-{\bar{z}}_{board}\right)}^{2}=\frac{1}{m-1}\sum_{k=1}^{m}{\left(\frac{1}{L_{m}\left(k\right)}\sum_{i=1}^{L_{m}\left(k\right)}(z_{i}-{\bar{z}}_{board}\right)}^{2})$$

Furthermore, the $\sigma_{board}^{2}$ of a whole is obtained according to

(13) $$\sigma_{board}^{2}=L_{m}\sigma_{i}^{2}=\frac{L_{m}}{\left(m-1\right)L_{m}}\sum_{i=1}^{m}\sigma_{m}^{2}\left(i\right)=\frac{1}{m-1}\sum_{i=1}^{m}\sigma_{m}^{2}\left(i\right)$$

As a result, the proof is completed.

The lemma shows that the performance of the board can be obtained through the combination of different groups. For a board tunnel, this means the total probability of healthy model can be combined with different input signals.

## 3. The Proposed Approach

### 3.1. Probability Neural Network

The probability neural network (PNN) that was proposed by D.F Specht in 1990 is a kind of statistical neural network model based on a Bayesian minimum risk criterion [24]. It consists of four layers, including the input layer, the pattern layer, the summation layer, and the output layer. The input layer is responsible for transmitting the feature vector into the network. The pattern layer takes full connection directly from the input layer through the connection weight. The pattern layer reflects the spatial distribution of the samples, in which each sample works in a limited local space, and the whole space constitutes a distributed probability distribution with a sample combination. This structure accurately reflects the probability distribution of the sample in the whole space. It is usually trained with supervised learning based on training samples and the responding patterns. The distance between the input eigenvector and the trained pattern is used to activate the Gaussian function of the pattern layer. The summation layer is responsible for connecting the outputs of the pattern layer and the schema units of each class through the score probability. Finally, the output layer outputs the category with the highest total score of schema units of each class in the summation layer. In PNN, the probability density $p\left(x|w_{i}\right)$ is expressed by a radial basis function:

(14) $$p\left(x|w_{i}\right)=\frac{1}{N_{i}}\sum_{k=1}^{N_{i}}\frac{1}{2\pi^{\frac{l}{2}}\sigma^{l}}\exp\left(-\frac{{\left|x-x_{ik}\right|}^{2}}{2\sigma^{2}}\right)$$

where $x_{ik}$,$N_{i}$, σ, and l are the sample center, the smoothness factor, the hyper-parameters, and the coefficient, respectively. The discriminant function $g_{i}\left(x\right)$ is

(15) $$g_{i}\left(x\right)=\frac{p\left(w_{i}\right)}{N_{i}}\sum_{k=1}^{N_{i}}\exp\left(-\frac{{\left|x-x_{ik}\right|}^{2}}{2\sigma^{2}}\right)=\frac{p\left(w_{i}\right)}{N_{i}}\sum_{k=1}^{N_{i}}\exp\left(-\frac{x^{T}x_{ik}-1}{\sigma^{2}}\right)$$

where $p\left(w_{i}\right)$ is the probability of $w_{i}$ occurrence.

Additionally, the discrimination rule is

(16) $$\mathrm{if}\;g_{i}\left(x\right)>g_{j}\left(x\right)\forall i\neq j,\mathrm{then}x\in w_{i}$$

### 3.2. The Construction of Critical Faulty Data

**Figure 3:**
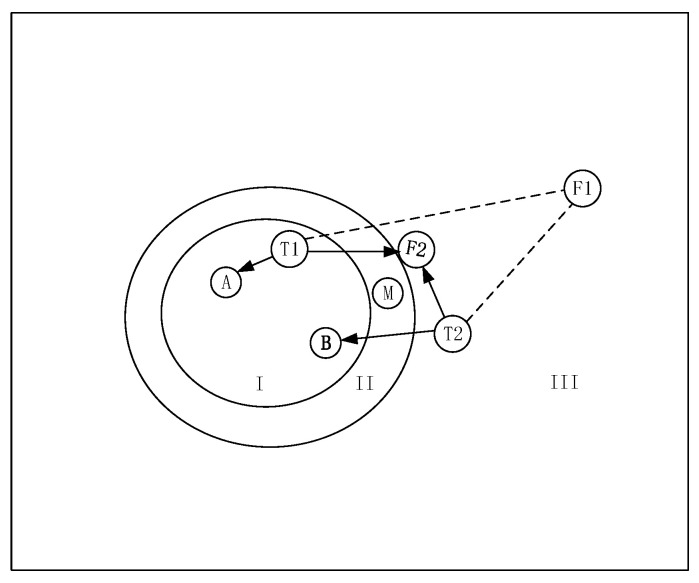
The schematic diagram of critical faulty data construction.

The PNN distinguishes the category of input data based on the established relationship of the train examples and the category belonged to. Different from the weights principle of direct mapping between input and output, the PNN adopts computing the proximity to the different sample data and judges the category according to a posterior probability. In principle, as long as there are faulty data samples and health data samples, the new data will be classified in healthy states and faulty states, except for an occurrence of posteriori probability of just 50%. However, the fault samples are in fact in a large range that affects the accuracy as a criterion. The schematic diagram of critical faulty data construction is shown in Figure 3.

In Figure 3, the square represents the entire set of healthy and faulty states, which is classed as section I (health), section II (vagueness), and section III (fault). The A and the B are the observed sets that build the health data samples. The F1 and the F2 are the faulty data samples. Additionally, the T1 and the T2 are the test sets. For a healthy dataset T1, it is prone to find an observed health set A that is close to T1. However, for a faulty dataset T2, if one randomly selects the faulty dataset F1 as faulty data samples, it will produce the incorrect result that the T2 is health because the distance from T2 to B is closer than that from T2 to F1. If the position of F1 moves to the position of F2 that belongs to section III but is close to section II, the previous mistakes will be avoided. Thus, the fault samples at the edge of vagueness and fault are called critical faulty data. Although the critical faulty data cannot distinguish the dataset of all sections (for example, the M of section II), they can solve the judgment problem for the most of the datasets.

However, the board channel has almost no historical faulty data to be used because the board channel is prohibited from working with faults. This makes it impossible to find the critical faulty data by analyzing historical data. To produce the examples of critical faulty data from the healthy data, the null matrix is introduced as a vertical cross mode of the healthy state and the critical faulty data. The null matrix N of a non-full rank matrix X is defined if there is a matrix N that satisfies XN = 0 and NN = I [25].

According to the definition of the null matrix, for $x_{i}$ being a sampling vector of healthy data, there is

(17) $$N_{i}x_{i}=0$$

where $N_{i}$ is the corresponding null matrix.

For another sampling vector $x_{j}$ ($x_{j}\neq x_{i}$), there is

(18) $$N_{i}x_{j}=b_{ij}$$

where $b_{ij}$ is the deviation of $x_{j}$ under the action of null matrix $N_{i}$.

Compute the deviation $b_{kl}$ of all samples $x_{l}\left(l=1\ldots n\right)$ and null matrix $N_{k}\left(k=1\ldots n\right)$ according to

(19) $$b_{kl}=N_{k}x_{l}\left(k=1.n;L=1\ldots N\right)$$

Take $b=max\left\{b_{kl},k=1..n;l=1\ldots n\right\}$ and obtain the corresponding null matrix N for all healthy data, and inequality (20) is satisfied:

(20) Nx≤b

The corresponding equation reflects the critical state of fault and health:

(21) Nx=b

Move the left of Formula (21) to the right and replace I with $NN^{-1}$:

(22) $$Nx-NN^{-1}b=0$$

where $N^{-1}$ is a pseudo inverse of N.

We obtain

(23) $$N\left(x-N^{-1}b\right)=0$$

Let

(24) $$\overset{´}{x}=x-N^{-1}b$$

and $\overset{´}{x}$ is the critical faulty data.

### 3.3. The Structure and Workflow of Proposed Approach

**Figure 4:**
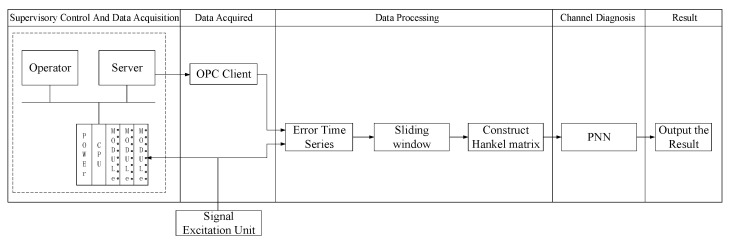
The structure of proposed approach.

The proposed method is made up with four parts, including the data acquisition, the data processing, the probability neural network, and the diagnostic output. The excitation source acts on the board channel with multiple groups of different kinds of signals in order to expand the detection scope as much as possible. The error time series is built from the excitation signal and the converted data by a technique of OLE for process control (OPC) [26]. Then, it is transformed to a Hankel matrix by a sliding window in order to adapt to the PNN training. The diagnostic result is output by the PNN. The structure is shown in Figure 4.

The workflow is described as follows:

Step 1: Record the signal generator and use OPC to obtain the internal memory data of the board tunnel. Thus, the error time series ${\left\{z_{k}\right\}}_{k=1}^{j}$ combined with different input signals is formed according to Formula (1).

Step 2: Suppose the length of the sliding window is T, and construct the Hankel matrix $H_{L}$ with depth L (usually L≫T):

(25) $$H_{L}={\left[\begin{array}{c} \begin{array}{cc} z_{l} & \begin{array}{ccc} z_{l+1} & \cdots & z_{l+T-L} \end{array} \end{array}\\ \begin{array}{cc} z_{l+1} & \begin{array}{ccc} z_{l+2} & \cdots & z_{l+1+T-L} \end{array} \end{array}\\ \begin{array}{c} \begin{array}{cc} \vdots \; & \begin{array}{ccc} \vdots \; & \ddots \; & \vdots \; \end{array} \end{array}\\ \begin{array}{cc} z_{l+L-1} & \begin{array}{ccc} z_{l+T} & \cdots & z_{l+T-1} \end{array} \end{array} \end{array} \end{array}\right]}_{L\times T}$$

Step 3: The critical faulty dataset *H_LN_* is constructed according to Formula (24) of 3.2:

(26) $$H_{LN}={\left[\begin{array}{c} \begin{array}{cc} {\overset{´}{z}}_{l} & \begin{array}{ccc} {\overset{´}{z}}_{l+1} & \cdots & {\overset{´}{z}}_{l+T-L} \end{array} \end{array}\\ \begin{array}{cc} {\overset{´}{z}}_{l+1} & \begin{array}{ccc} {\overset{´}{z}}_{l+2} & \cdots & {\overset{´}{z}}_{l+1+T-L} \end{array} \end{array}\\ \begin{array}{c} \begin{array}{cc} \vdots & \begin{array}{ccc} \vdots \; & \ddots \; & \vdots \; \end{array} \end{array}\\ \begin{array}{cc} {\overset{´}{z}}_{l+L-1} & \begin{array}{ccc} {\overset{´}{z}}_{l+T} & \cdots & {\overset{´}{z}}_{l+T-1} \end{array} \end{array} \end{array} \end{array}\right]}_{L\times T}$$

Step 4: Construct the sample matrix of the PNN by using input H:

(27) $$\text{H}={\left[H_{L}\;H_{LN}\right]}_{L\times 2T}={\left[\;\begin{array}{cccccccc} z_{l} & z_{l+1} & \cdots & z_{l+T-L} & {\overset{´}{z}}_{l} & {\overset{´}{z}}_{l+1} & \cdots & {\overset{´}{z}}_{l+T-L}\\ z_{l+1} & z_{l+2} & \cdots & z_{l+1+T-L} & {\overset{´}{z}}_{l+1} & {\overset{´}{z}}_{l+2} & \cdots & {\overset{´}{z}}_{l+1+T-L}\\ \vdots \; & \vdots \; & \ddots \; & \vdots \; & \vdots \; & \vdots \; & \ddots \; & \vdots \;\\ z_{l+L-1} & z_{l+T} & \cdots & z_{l+T-1} & {\overset{´}{z}}_{l+L-1} & {\overset{´}{z}}_{l+T} & \cdots & {\overset{´}{z}}_{l+T-1} \end{array}\right]}_{L\times 2T}$$

Moreover, the corresponding category is [0 1], where 0 and 1 represent the healthy states and the faulty states, respectively.

Step 5: Build the PNN by following three rules: (1) the number of input layers is the length of the sliding window $\left(T\right)$; (2) the number of neurons in the mode layer is the number of input sample vectors $\left(L\right)$; and (3) the summation layer is of class 2, which represents health and fault.

Step 6: The test sequence ${\left\{T_{k}\right\}}_{k=1}^{j}$ is converted to the input sample matrix D by normalizing the Hankel matrix, denoted as

(28) $$D=\text{Norm}{\left[\begin{array}{cccccccc} T_{l} & T_{l+1} & \cdots & T_{l+T-L} & {\overset{´}{T}}_{l} & {\overset{´}{T}}_{l+1} & \cdots & {\overset{´}{T}}_{l+T-L}\\ T_{l+1} & T_{l+2} & \cdots & T_{l+1+T-L}\; & {\overset{´}{T}}_{l+1} & {\overset{´}{T}}_{l+2} & \cdots & {\overset{´}{T}}_{l+1+T-L}\\ \vdots \;\;\; & \vdots \;\; & \ddots \;\;\; & \vdots \;\; & \vdots \;\;\; & \vdots \;\; & \ddots \;\;\; & \vdots \;\;\\ T_{l+L-1} & T_{l+T} & \cdots & T_{l+T-1} & {\overset{´}{T}}_{l+L-1} & {\overset{´}{T}}_{l+T} & \cdots & {\overset{´}{T}}_{l+T-1} \end{array}\right]}_{L\times 2T}\;={\left[\begin{array}{cccc} D_{11} & D_{12} & \cdots & D_{1,2T}\\ D_{21} & D_{22} & \cdots & D_{2,2T}\\ \vdots & \vdots & \ddots & \vdots \\ D_{q1} & D_{q2} & \cdots & D_{q,2T} \end{array}\right]}_{q\times 2T}$$

The sample reference C is obtained by row normalizing the train of input matrix H

(29) $$C=\text{Norm}{\left[\begin{array}{cccccccc} Z_{l} & Z_{l+1} & \cdots & Z_{l+T-L} & {\overset{´}{Z}}_{l} & {\overset{´}{Z}}_{l+1} & \cdots & {\overset{´}{Z}}_{l+T-L}\\ Z_{l+1} & Z_{l+2} & \cdots & Z_{l+1+T-L}\; & {\overset{´}{Z}}_{l+1} & {\overset{´}{Z}}_{l+2} & \cdots & {\overset{´}{Z}}_{l+1+T-L}\\ \vdots \;\;\; & \vdots \;\; & \ddots \;\;\; & \vdots \;\; & \vdots \;\;\; & \vdots \;\; & \ddots \;\;\; & \vdots \;\;\\ Z_{l+L-1} & Z_{l+T} & \cdots & Z_{l+T-1} & {\overset{´}{Z}}_{l+L-1} & {\overset{´}{Z}}_{l+T} & \cdots & {\overset{´}{Z}}_{l+T-1} \end{array}\right]}_{L\times 2T}\;={\left[\begin{array}{cccc} C_{11} & C_{12} & \cdots & C_{1,2T}\\ C_{21} & C_{22} & \cdots & C_{2,2T}\\ \vdots & \vdots & \ddots & \vdots \\ C_{l1} & C_{l2} & \cdots & C_{l,2T} \end{array}\right]}_{L\times 2T}$$

where the $\mathrm{Norm}\left[∙\right]$ is an operator of matrix row normalizing.

Step 7: Calculate the Euclidean distance between the input matrix D and the sample reference matrix C according to


(30) $$\text{E}={\left[\begin{array}{cccc} \sqrt{\overset{2T}{\underset{j=1}{\sum }}{\left|D_{1j}-C_{1j}\right|}^{2}} & \sqrt{\overset{2T}{\underset{j=1}{\sum }}{\left|D_{1j}-C_{2j}\right|}^{2}} & \cdots & \sqrt{\overset{2T}{\underset{j=1}{\sum }}{\left|D_{1j}-C_{lj}\right|}^{2}}\\ \sqrt{\overset{2T}{\underset{j=1}{\sum }}{\left|D_{2j}-C_{1j}\right|}^{2}} & \sqrt{\overset{2T}{\underset{j=1}{\sum }}{\left|D_{2j}-C_{2j}\right|}^{2}} & \cdots & \sqrt{\overset{2T}{\underset{j=1}{\sum }}{\left|D_{2j}-C_{Tj}\right|}^{2}}\\ \vdots \; & \vdots \; & \ddots \; & \vdots \\ \sqrt{\overset{2T}{\underset{j=1}{\sum }}{\left|D_{qj}-C_{1j}\right|}^{2}} & \sqrt{\overset{2T}{\underset{j=1}{\sum }}{\left|D_{qj}-S_{2j}\right|}^{2}} & \cdots & \sqrt{\overset{2T}{\underset{j=1}{\sum }}{\left|D_{qj}-S_{Tj}\right|}^{2}} \end{array}\right]}_{q\times 2T}\;={\left[\begin{array}{cccc} E_{11} & E_{12} & \cdots & E_{1,2T}\\ E_{21} & E_{22} & \cdots & E_{2,2T}\\ \vdots & \vdots & \ddots & \vdots \\ E_{q1} & E_{q2} & \cdots & E_{q,2T} \end{array}\right]}_{q\times 2T}$$

Step 8: The initial probability matrix P is obtained by activating the Gaussian function of the pattern layer:

(31) $$\text{P}={\left[\begin{array}{cccc} e^{-\frac{E_{11}}{2\sigma^{2}}} & e^{-\frac{E_{12}}{2\sigma^{2}}} & \cdots & e^{-\frac{E_{1,T}}{2\sigma^{2}}}\\ e^{-\frac{E_{21}}{2\sigma^{2}}} & e^{-\frac{E_{22}}{2\sigma^{2}}} & \cdots & e^{-\frac{E_{2,T}}{2\sigma^{2}}}\\ \vdots & \vdots & \ddots & \vdots \\ e^{-\frac{E_{q1}}{2\sigma^{2}}} & e^{-\frac{E_{q2}}{2\sigma^{2}}} & \cdots & e^{-\frac{E_{q,T}}{2\sigma^{2}}} \end{array}\right]}_{q\times 2T}={\left[\begin{array}{cccc} P_{11} & P_{12} & \cdots & P_{1,T}\\ P_{21} & P_{22} & \cdots & P_{2,T}\\ \vdots & \vdots & \ddots & \vdots \\ P_{q1} & P_{q2} & \cdots & P_{q,T} \end{array}\right]}_{q\times 2T}$$

Step 9: The probability S that q samples belong to two categories (health and fault) is obtained according to Formula (29):

(32) $$\text{S}={\left[\begin{array}{cc} \overset{T}{\underset{j=1}{\sum }}P_{1j} & \overset{2T}{\underset{j=T+1}{\sum }}P_{1j}\\ \overset{T}{\underset{j=1}{\sum }}P_{2j} & \overset{2T}{\underset{j=T+1}{\sum }}P_{2j}\\ \cdots & \cdots \\ \overset{T}{\underset{j=1}{\sum }}P_{qj} & \overset{2T}{\underset{j=T+1}{\sum }}P_{qj} \end{array}\right]}_{q\times 2}={\left[\begin{array}{cc} S_{11} & S_{21}\\ S_{21} & S_{22}\\ \cdots & \cdots \\ S_{q1} & S_{q2} \end{array}\right]}_{q\times 2}$$

Step 10: The maximum probability of each row is taken as the category according to Bayesian decision theory.

## 4. Case Studies

**Figure 5:**
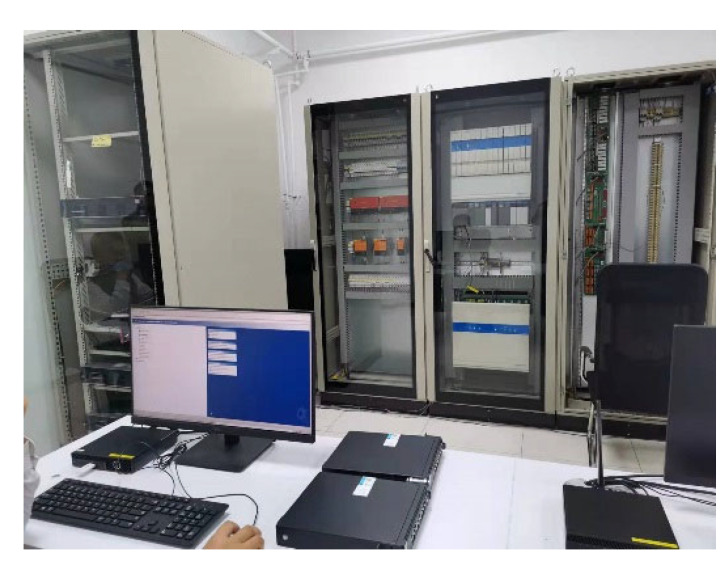
The central control platform.

The experimental platform is a distributed control system with an engineer station. Our goal was to test the performance of the board without any destruction. The input signal of the board tunnel for the test was imposed directly by another board tunnel because the board channel of the laboratory is not loaded. If the board channel is connected to the sensor signal, it will reform the input signal by adding a small series signal source (usually not more than 15% of the normal signal amplitude). This small series signal is used only to detect the performance of the board tunnel and is easily eliminated by software. The central control platform of the laboratory is shown in Figure 5.

There were five groups of healthy data with input signals of 5 V with additional pulse voltage, piecewise linear voltage, exponential voltage, thermal noise, and chirp signal. However, it was a challenge to construct any faults of the board tunnel because the board was not to be disassembled or damaged. Losing faults by changing internal states, there is only one possibility that uses the calibration function of the control system to change the AD converted reference signal. Two groups of faulty data were simulated by changing the AD converted reference signal with the addition of the stochastic disturbance and the periodic voltage, respectively. The cases of seven groups are shown in Table 1.

### 4.1. Change the Number of Intermediate Layers of PNN

Eight numbers of sliding window length from 100 to 20,000 were selected to detect the state of case5 to case 7. It repeated 1000 times per sliding window length. The training samples were combined with case1, case2, case3, and case4. For each test, the starting of sliding window was randomly selected from the error time series, and the Hankel depth was always kept at 10,000, just for simplicity. The results are shown in Table 2.

It is seen from Table 2 that the lengths of the sliding window have an effect on the detection results. Short lengths of 100 samples and 150 samples succeeded in detecting the healthy states but failed to find the faulty states by mistaking them for the healthy states. With the lengths expanding to 200 samples and 500 samples, the accuracy of fault detection increased by more than 99% (case6 and case7 achieve 99.9% and 99.2%, respectively), although the accuracy of the healthy states was reduced to 89.6% and 89.7%. When the length of the sliding window reached 2000 samples, the detection of healthy states and two faulty states could reach 100%. However, it is not that the longer the sliding window length is, the better the result is. When the window length reached 20,000 samples, the detection results were all healthy states regardless of healthy states or fault status. In other words, the faulty states could not be determined at sliding window lengths that were too long.

### 4.2. Effects of Different Groups of Health Data Combination as Sample Input

The combination of four groups of health data was selected as the training samples to detect the fifth group of healthy states and the other groups of two faulty states. The length of the sliding window was 2000 samples, and the depth of the Hankel was 10,000. A test was done according to the proposed method, and the results are shown in Table 3, which indicates an accuracy of 100%, whether healthy or faulty.

A change of combination from four groups to three groups of healthy data was used to test the effects of training samples from different group combinations. The length of the sliding window and the depth of the Hankel were kept unchanged. The results are shown in Table 4.

Table 4 shows that the most health and faults can be detected by combining three groups of health data as training examples via the proposed method. However, a few health cases are in states of poor accuracy because the training examples can partly cover the information of other health cases. This will be further confirmed by reducing the number of groups for training examples. In the cases of taking two groups as a combination of training examples, the situation is similar to before. Most health and faults can be detected correctly, but there are some incorrect detection results for healthy states. For example, taking case1 and case5 as training examples, the results of case2 and case4 are correct, but the results of case3 are all wrong in 1000 tests. These results are not listed here due to space limitations. By analyzing the above situations, we found that incorrect detection is related to some kinds of healthy data. It is due to the reason that the training data do not completely cover the characteristics of the test samples. We also notice that the detection results for faulty states are correct, which shows that the null matrix plays an important role. A conclusion is drawn that the feature coverage of training samples is more important than the number of groups.

### 4.3. Comparison with LDM

The classical linear discriminative method (LDM) was used to detect the fault of board channel. The 10,000 groups combined from the time series were selected as training samples whose length of the sliding window was 2000 samples, and a random 1000 groups of each case were tested for imitating the situation with known historical data. The results are shown in Table 5.

The 1000 groups of data from case7 were used be tested as unknown faulty data, and the results are shown in Table 6.

It is seen from Table 5 that for the labeled data, the LDM has a high accuracy of more than 99.3%, and it can be divided into more detailed categories. However, Table 6 shows that the accuracy of the LDM for a new fault is 70.3%, which is low. Compared with the LDM, the proposed approach, which is shown in Table 3, can achieve good results only by using healthy data.

## 5. Conclusions

At present, there is no practical method to detect the enclosed board tunnel except for returning it to the factory or an error code display. Failure to find the abnormal board brings a great potential threat to the control system of the plant. This paper proposes an approach for fault detection of an enclosed board channel by using a PNN based on an error time series excited by various external signals. The critical faulty data, contrary to the known healthy data, are constructed by using a null matrix with maximum projection and are labelled as training examples together with healthy data. This provides the mode criteria of PNN training. Thus, the problem of PNN lacking faulty data examples has been solved to some extent. The proposed approach is a data-driven method that can detect the abnormal state or fault of an enclosed board channel without knowing any internal circuit of the board channel. It only needs a small number of additional hardware devices and does not need any mechanism knowledge on the board channel, which greatly reduces the costs and the professional knowledge for staff. In the future, cases where the output probabilities of the health mode and fault mode are similar will be studied, which should improve the accuracy of the proposed approach in some special scenarios.

## Figures and Tables

**Table 1:** The cases of 7 groups of signals.

No.	Symbols	Description
1	Case1	Input signal with additional pulse voltage of duty cycle 50% and frequency 20 Hz
2	Case2	Input signal with additional piecewise linear voltage of slope 0.5; amplitude: 0 to −2 V
3	Case3	Input signal with additional exponential voltage from 0 to 2 V in 5 s
4	Case4	Input signal with additional thermal noise of 1 MHz bandwidth
5	Case5	Input signal with additional chirp signal: initial frequency—0 Hz; final frequency—500 Hz; amplitude—1 V; delay—0.05 s
6	Case6	The reference signal with additional random noise (Fault1)
7	Case7	The reference signal with periodic voltage signal (Fault2)

**Table 2:** Effect of sliding window length on detection results.

No.	Length of Sliding Window	Case5		Case6		Case7	
		Correct/Wrong (Times)	Accuracy	Correct/Wrong (Times)	Accuracy	Correct/Wrong (Times)	Accuracy
1	100	1000/0	100%	0/1000	0%	0/1000	0%
2	150	1000/0	100%	0/1000	0%	0/1000	0%
3	200	896/104	89.6%	999/1	99.9%	992/8	99.2%
4	500	897/103	89.7%	998/2	99.8%	971/29	97.1%
5	1000	922/78	92.2%	1000/0	100%	1000/0	100%
6	1500	905/95	90.5%	1000/0	100%	1000/0	100%
7	2000	1000/0	100%	1000/0	100%	1000/0	100%
8	20,000	1000/0	100%	0/1000	0%	0/1000	0%

**Table 3:** The combination of four groups of health data.

No.	Training Examples	Test	Correct (Times)	Wrong (Times)	Accuracy
1	Case1/Case2/Case3/Case4	Case5	1000	0	100%
		Case6	1000	0	100%
		Case7	1000	0	100%
2	Case1/Case2/Case3/Case5	Case4	1000	0	100%
		Case6	1000	0	100%
		Case7	1000	0	100%
3	Case1/Case2/Case4/Case5	Case3	1000	0	100%
		Case6	1000	0	100%
		Case7	1000	0	100%
4	Case1/Case3/Case4/Case5	Case2	1000	0	100%
		Case6	1000	0	100%
		Case7	1000	0	100%
5	Case2/Case3/Case4/Case5	Case1	1000	0	100%
		Case6	1000	0	100%
		Case7	1000	0	100%

**Table 4:** The combination of three groups of health data.

No.	Training Examples	Test	Correct (Times)	Wrong (Times)	Accuracy
1	Case1/Case2/Case3	Case4	1000	0	100%
		Case5	1000	0	100%
		Case6	1000	0	100%
		Case7	1000	0	100%
2	Case1/Case2/Case4	Case3	1000	0	100%
		Case5	1000	0	100%
		Case6	1000	0	100%
		Case7	1000	0	100%
3	Case1/Case2/Case5	Case3	1000	0	100%
		Case4	1000	0	100%
		Case6	1000	0	100%
		Case7	1000	0	100%
4	Case1/Case3/Case4	Case2	687	314	68.7%
		Case5	1000	0	100%
		Case6	1000	0	100%
		Case7	1000	0	100%
5	Case1/Case3/Case5	Case2	680	320	68%
		Case4	1000	0	100%
		Case6	1000	0	100%
		Case7	1000	0	100%
6	Case1/Case4/Case5	Case2	667	333	66.7%
		Case3	1000	0	100%
		Case6	1000	0	100%
		Case7	1000	0	100%
7	Case2/Case3/Case4	Case1	879	121	87.9%
		Case5	1000	0	100%
		Case6	1000	0	100%
		Case7	1000	0	100%
8	Case2/Case3/Case5	Case1	792	208	79.2%
		Case4	1000	0	100%
		Case6	1000	0	100%
		Case7	1000	0	100%
9	Case2/Case4/Case5	Case1	811	189	81.1%
		Case3	1000	0	100%
		Case6	1000	0	100%
		Case7	1000	0	100%
10	Case3/Case4/Case5	Case1	184	816	18.4%
		Case2	762	238	76.2%
		Case6	998	2	99.8%
		Case7	1000	0	100%

**Table 5:** The results of CNN for labeled data.

	Case1	Case2	Case3	Case4	Case5	Case6
Correct	993	1000	1000	1000	997	1000
Incorrect	7	0	0	0	13	0
Accuracy	99.3%	100%	100%	100%	99.7%	100%

**Table 6:** The results of CNN for unlabeled data.

	Health					Fault
	Case1	Case2	Case3	Case4	Case5	Case6
Case7	164	133	0	0	0	703
	Total: 297					

## Data Availability

Not applicable.
